# Supplementary material for: Risk Factors for Cancer Mortality in Spain: Population-Based Cohort Study
Source: Int J Environ Res Public Health. 2022 Aug 10;19(16):9852. doi: 10.3390/ijerph19169852 (PMC9408698; doi:10.3390/ijerph19169852)
Supplement: Supplementary file 1 [file ijerph-19-09852-s001.zip › ijerph-1843538-supplementary.pdf]

Table S1. All characteristics of the sample

		n	%
Mortality	Survived	19290	94.6%
	Death due to neoplasm	340	1.7%
	Death from other cause	767	3.8%
Autonomous communities	Andalusia	3659	17.9%
	Aragón	588	2.9%
	Asturias	485	2.4%
	Balearic Islands	477	2.3%
	Canary Islands	931	4.6%
	Cantabria	263	1.3%
	Castilla y León	1143	5.6%
	Castilla-La Mancha	918	4.5%
	Catalonia	3068	15.0%
	Valencian Community	2233	10.9%
	Extremadura	481	2.4%
	Galicia	1254	6.1%
	Madrid	2804	13.7%
	Murcia	649	3.2%
	Navarra	278	1.4%
	Basque Country	961	4.7%
	Rioja	141	0.7%
	Ceuta-Melilla	62	0.3%
Age group (years)	< 50	12015	58.9%
	50-59	3106	15.2%
	60-69	2393	11.7%
	70-79	1767	8.7%
	≥ 80	1115	5.5%
Sex	Male	9942	48.7%
	Female	10455	51.3%
Social Class	Social Class I	2185	10.7%
	Social Class II	1498	7.3%
	Social Class III	3646	17.9%
	Social Class IV	3046	14.9%
	Social Class V	6482	31.8%
	Social Class VI	3003	14.7%
	NA	538	2.6%
Body mass index	Normal (< 25 kg/m^2)	9019	44.2%
	Overweight (25.0 to < 30 kg/m^2)	6803	33.4%
	Obese (≥ 30 kg/m^2)	3144	15.4%
	NA	1431	7.0%
Country of Birth	Spain	17470	85.7%
	Abroad	2927	14.3%
Civil status	Single	6573	32.2%
	Married	11398	55.9%
	Widower	1460	7.2%
	Separated	364	1.8%
	Divorced	604	3.0%
Educational level	University	3280	16.1%
	Professional training	3126	15.3%
	Bachelor	2680	13.1%
	Secondary	6848	33.6%
	Primary or less	4462	21.9%
Net monthly income	NA	5441	26.7%
	> 2251 €	3195	15.7%
	1551-2250 €	3206	15.7%
	1051-1550 €	3765	18.5%
	801-1050 €	2077	10.2%
	< 800 €	2713	13.3%
Tobacco use	Never smoker	10908	53.5%
	Ex-smoker	3971	19.5%
	Smoker	5518	27.1%
Exposure to tabacco	Never	16250	79.7%

	< 1h / day	1567	7.7%
	> 1h / day	2581	12.7%
Alcohol intake	None	10857	53.2%
	Low risk (≤ 20 g/week women and ≤ 40 g/week men)	9029	44.3%
	Risky drinking (> 20 g/week women and > 40 g/week men)	511	2.5%
Hours of sleep	> 9h / day	1226	6.0%
	7-9 h / day	14643	71.8%
	< 7 h / day	4528	22.2%
Main daily activity	Sitting for the most part	8161	40.0%
	Standing for the most part	9199	45.1%
	Tasks with some effort	3038	14.9%
Physical activity	Sedentary	9051	44.4%
	Occasional physical activity	6735	33.0%
	Frequent physical activity	2668	13.1%
	Athletic training	1943	9.5%
Breakfast	At home	17288	84.8%
	Away from home	2335	11.4%
	I don't usually eat breakfast	774	3.8%
Fruit intake	Daily	12427	60.9%
	≥ 3 servings a week	3957	19.4%
	1-2 servings a week	2274	11.1%
	< 1 serving a week	787	3.9%
	Rarely or never	953	4.7%
Vegetable intake	Daily	9566	46.9%
	≥ 3 servings a week	6990	34.3%
	1-2 servings a week	2761	13.5%
	< 1 serving a week	690	3.4%
	Rarely or never	390	1.9%
Intake of legumes	Daily	312	1.5%
	≥ 3 servings a week	4738	23.2%
	1-2 servings a week	12161	59.6%
	< 1 serving a week	2400	11.8%
	Rarely or never	786	3.9%
Dairy intake	Daily	17231	84.5%
	≥ 3 servings a week	1557	7.6%
	1-2 servings a week	702	3.4%
	< 1 serving a week	348	1.7%
	Rarely or never	559	2.7%
Sweets intake	Daily	5924	29.0%
	≥ 3 servings a week	3105	15.2%
	1-2 servings a week	3708	18.2%
	< 1 serving a week	3278	16.1%
	Rarely or never	4382	21.5%
Fast food	Daily	360	1.8%
	≥ 3 servings a week	919	4.5%
	1-2 servings a week	4078	20.0%
	< 1 serving a week	4861	23.8%
	Rarely or never	10180	49.9%
Dental hygiene	3 o more times a day	7758	38.0%
	2 times / day	6738	33.0%
	1 time / day	4034	19.8%
	Never or ocasionally	1866	9.1%
Self-perceived health	Very good	4396	21.6%
	Good	10413	51.0%
	Okay	4065	19.9%
	Bad	1224	6.0%
	Very bad	299	1.5%
EuroQol-5D EVA	> 75 points	13425	65.8%
	50-75 points	5401	26.5%
	< 50 points	1570	7.7%
Some chronic illness	No	11823	58.0%

	Yes	8574	42.0%
Hypertension	No	16119	79.0%
	Yes	4278	21.0%
Myocardial infarction	No	19997	98.0%
	Yes	400	2.0%
Other heart diseases	No	19226	94.3%
	Yes	1171	5.7%
Varicose veins	No	18087	88.7%
	Yes	2310	11.3%
Arthrosis or arthritis	No	16663	81.7%
	Yes	3734	18,3%
Chronic neck pain	No	17053	83.6%
	Yes	3344	16.4%
Chronic low back pain	No	16427	80.5%
	Yes	3970	19.5%
Chronic allergy	No	17921	87.9%
	Yes	2476	12.1%
Allergic asthma	No	19359	94.9%
	Yes	1038	5.1%
COPD	No	19459	95.4%
	Yes	938	4.6%
Diabetes mellitus	No	18932	92.8%
	Yes	1465	7.2%
Gastrointestinal ulcer	No	19545	95.8%
	Yes	852	4.2%
Urinary incontinence	No	19710	96.6%
	Yes	687	3.4%
High cholesterol	No	16617	81.5%
	Yes	3780	18.5%
Waterfalls	No	18779	92.1%
	Yes	1618	7.9%
Skin problems	No	19432	95.3%
	Yes	965	4.7%
Constipation	No	19572	96.0%
	Yes	825	4.0%
Cirrhosis	No	20195	99.0%
	Yes	202	1.0%
Depression	No	18918	92.7%
	Yes	1479	7.3%
Anxiety	No	18836	92.3%
	Yes	1561	7.7%
Mental health problems	No	20109	98.6%
	Yes	288	1.4%
Stroke	No	20163	98.9%
	Yes	234	1.1%
Migraine	No	18419	90.3%
	Yes	1978	9.7%
Hemorrhoids	No	19200	94.1%
	Yes	1197	5.9%
Malignant tumor	No	19822	97.2%
	Yes	575	2.8%
Osteoporosis	No	19570	95.9%
	Yes	827	4.1%
Thyroid problems	No	19324	94.7%
	Yes	1073	5.3%
Accident injuries	No	19300	94.6%
	Yes	1097	5.4%
Accidents in the last year	No	18699	91.7%
	Yes	1698	8.3%
Mental health	Psychopathology absence	17796	87.2%
	Suspicion	994	4.9%
	Psychopathology presence	1367	6.7%

	NA	240	1.2%
Limitation of physical activity in previous 2 weeks	No	18077	88.6%
	Yes	2320	11.4%
Bed rest in previous 2 weeks	No	19297	94.6%
	Yes	1100	5.4%
Limitation of physical activity in previous 6 months	Severely limited	672	3.3%
	Limited not serious	3238	15.9%
	Nothing limited	16487	80.8%
Glasses or contact lenses	No	7953	39.0%
	Yes	12444	61.0%
Hearing aids	No	19764	96.9%
	Yes	633	3.1%
Hospitalization in previous 12 months	No	18702	91.7%
	Yes	1695	8.3%
Primary care consult in previous month	No	14678	72.0%
	Yes	5719	28.0%
Visits to other specialists	No	17330	85.0%
	Yes	3067	15.0%
Stay in day hospital in previous 12 months	No	18858	92.5%
	Yes	1539	7.5%
Visits to urgencies in previous 12 months	No	14926	73.2%
	Yes	5471	26.8%
Physiotherapist in previous 12 months	No	18297	89.7%
	Yes	2100	10.3%
Psychologist in previous 12 months	No	19700	96.6%
	Yes	697	3.4%
Radiography in previous 12 months	No	14831	72.7%
	Yes	5566	27.3%
CT or scanner in previous 12 months	No	18861	92.5%
	Yes	1536	7.5%
Ecography in previous 12 months	No	17472	85.7%
	Yes	2925	14.3%
MRI in previous 12 months	No	18884	92.6%
	Yes	1513	7.4%
Flu vaccine	No	16610	81.4%
	Yes	3787	18.6%

CT: computed tomography; MRI: magnetic resonance imaging; NA: not available.

Table S2. Accumulated 6-year incidence rates for mortality from tumors and mortality from other causes according to demographic characteristics, other chronic pathologies, quality of life, and use of health services.

		Alive		Tumor mortality		Mortality other causes		
		n	%	n	%	n	%	p-value
Autonomous communities	Andalusia	3431	93.8%	68	1.9%	160	4.4%	0.043
	Aragón	553	94.0%	12	2.1%	23	3.9%	
	Asturias	447	92.1%	12	2.4%	27	5.5%	
	Balearic Islands	453	94.9%	3	0.6%	22	4.5%	
	Canary Islands	887	95.3%	10	1.1%	33	3.6%	
	Cantabria	251	95.4%	4	1.3%	9	3.3%	
	Castilla y León	1052	92.0%	29	2.6%	62	5.4%	
	Castilla-La Mancha	863	94.0%	16	1.7%	39	4.3%	
	Catalonia	2934	95.6%	46	1.5%	88	2.9%	
	Valencian Community	2118	94.8%	39	1.8%	76	3.4%	
	Extremadura	451	93.8%	11	2.2%	19	4.0%	
	Galicia	1190	94.9%	19	1.5%	45	3.6%	
	Madrid	2670	95.2%	40	1.4%	94	3.4%	
	Murcia	622	95.7%	6	0.9%	22	3.4%	
	Navarra	266	95.5%	6	2.0%	7	2.5%	
	Basque Country	911	94.8%	17	1.7%	34	3.5%	
	Rioja	132	93.8%	3	1.9%	6	4.3%	
	Ceuta-Melilla	59	96.3%	1	1.2%	2	2.5%	
Sex	Male	9349	94.0%	208	2.1%	386	3.9%	<0.001
	Female	9941	95.1%	132	1.3%	382	3.7%	
Age group (years)	< 50	11950	99.5%	23	0.2%	42	0.4%	<0.001
	50-59	3015	97.1%	59	1.9%	32	1.0%	
	60-69	2238	93.5%	71	3.0%	84	3.5%	
	70-79	1492	84.4%	89	5.0%	187	10.6%	
	≥ 80	595	53.3%	98	8.8%	423	37.9%	
Social Class	Social Class I	2091	95.7%	31	1.4%	63	2.9%	<0.001
	Social Class II	1439	96.1%	24	1.6%	35	2.3%	
	Social Class III	3466	95.1%	65	1.8%	115	3.1%	
	Social Class IV	2869	94.2%	62	2.0%	116	3.8%	
	Social Class V	6108	94.2%	102	1.6%	272	4.2%	
	Social Class VI	2823	94.0%	48	1.6%	132	4.4%	
	NA	496	92.2%	8	1.4%	35	6.4%	
Body mass index	Normal	8721	96.7%	103	1.1%	195	2.2%	<0.001
	Overweight	6450	94.8%	134	2.0%	220	3.2%	
	Obese	2922	92.9%	57	1.8%	165	5.2%	
	NA	1197	83.7%	46	3.2%	188	13.1%	
Country of Birth	Spain	16417	94.0%	316	1.8%	737	4.2%	<0.001
	Foreign	2873	98.2%	23	0.8%	31	1.1%	
Civil status	Single	6449	98.1%	34	0.5%	89	1.4%	<0.001
	Married	10810	94.8%	220	1.9%	367	3.2%	
	Widower	1098	75.2%	68	4.7%	293	20.1%	
	Separated	347	95.5%	8	2.2%	8	2.3%	
	Divorced	586	97.0%	8	1.4%	10	1.6%	
Educational level	University	3193	97.3%	37	1.1%	50	1.5%	<0.001
	Professional training	3074	98.3%	25	0.8%	28	0.9%	
	Bachelor	2608	97.3%	34	1.3%	38	1.4%	
	Secondary	6598	96.4%	81	1.2%	169	2.5%	
	Primary or less	3816	85.5%	163	3.6%	483	10.8%	
Net monthly income	NA	5174	95.1%	84	1.5%	183	3.4%	<0.001
	> 2251 €	3107	97.2%	33	1.0%	56	1.7%	
	1551-2250 €	3086	96.2%	37	1.2%	83	2.6%	
	1051-1550 €	3553	94.4%	65	1.7%	147	3.9%	
	801-1050 €	1943	93.6%	33	1.6%	100	4.8%	
	< 800 €	2426	89.4%	88	3.2%	199	7.3%	

Tobacco use	Never smoker	10280	94.3%	149	1.4%	478	4.4%	<0.001
	Ex-smoker	3652	92.0%	118	3.0%	201	5.1%	
	Smoker	5357	97.1%	72	1.3%	89	1.6%	
Exposure to tabacco	Never	15274	94.0%	296	1.8%	680	4.2%	<0.001
	< 1h / day	1508	96.3%	21	1.4%	37	2.4%	
	> 1h / day	2508	97.2%	22	0.9%	50	1.9%	
Alcohol intake	None	10124	93.2%	197	1.8%	536	4.9%	<0.001
	Low risk (≤ 20/40 g)	8679	96.1%	134	1.5%	216	2.4%	
	Risky drinking (> 20/40 g)	487	95.4%	8	1.6%	15	3.0%	
Hours of sleep	> 9h / day	1025	83.6%	39	3.2%	162	13.2%	<0.001
	7-9 h / day	14030	95.8%	204	1.4%	409	2.8%	
	< 7 h / day	4234	93.5%	97	2.1%	196	4.3%	
Main daily activity	Sitting for the most part	7351	90.1%	199	2.4%	610	7.5%	<0.001
	Standing for the most part	8958	97.4%	115	1.2%	126	1.4%	
	Tasks with some effort	2980	98.1%	26	0.8%	31	1.0%	
Physical activity	Sedentary	8267	91.3%	203	2.2%	581	6.4%	<0.001
	Occasional physical activity	6465	96.0%	119	1.8%	151	2.2%	
	Frequent physical activity	2641	99.0%	10	0.4%	17	0.6%	
	Athletic training	1916	98.6%	9	0.4%	18	0.9%	
Breakfast	At home	16243	94.0%	322	1.9%	723	4.2%	<0.001
	Away from home	2287	98.0%	12	0.5%	35	1.5%	
	I don't usually eat breakfast	760	98.1%	6	0.8%	9	1.1%	
Fruit intake	Daily	11655	93.8%	225	1.8%	547	4.4%	<0.001
	≥ 3 servings a week	3774	95.4%	64	1.6%	118	3.0%	
	1-2 servings a week	2185	96.1%	27	1.2%	61	2.7%	
	< 1 serving a week	761	96.8%	11	1.4%	15	1.9%	
	Rarely or never	914	95.9%	13	1.3%	26	2.7%	
Vegetable intake	Daily	9053	94.6%	143	1.5%	371	3.9%	0.188
	≥ 3 servings a week	6595	94.3%	135	1.9%	260	3.7%	
	1-2 servings a week	2622	95.0%	48	1.7%	91	3.3%	
	< 1 serving a week	657	95.2%	9	1.2%	24	3.5%	
	Rarely or never	363	93.0%	6	1.4%	22	5.5%	
Intake of legumes	Daily	298	95.5%	4	1.2%	10	3.3%	0.076
	≥ 3 servings a week	4449	93.9%	86	1.8%	203	4.3%	
	1-2 servings a week	11515	94.7%	200	1,6%	446	3.7%	
	< 1 serving a week	2290	95.4%	41	1.7%	69	2.9%	
	Rarely or never	737	93.8%	10	1.2%	39	5.0%	
Dairy intake	Daily	16281	94.5%	291	1.7%	659	3.8%	0.045
	≥ 3 servings a week	1487	95.5%	20	1.3%	50	3.2%	
	1-2 servings a week	677	96.3%	5	0.7%	21	2.9%	
	< 1 serving a week	328	94.2%	7	2.1%	13	3.7%	
	Rarely or never	517	92.5%	17	3.0%	25	4.5%	
Sweets intake	Daily	5587	94.3%	95	1.6%	243	4.1%	<0.001
	≥ 3 servings a week	2977	95.9%	36	1.2%	91	2.9%	
	1-2 servings a week	3532	95.3%	56	1,5%	120	3.2%	
	< 1 serving a week	3134	95.6%	55	1.7%	89	2.7%	
	Rarely or never	4060	92.6%	97	2.2%	225	5.1%	
Fast food	Daily	358	99.6%	0	0.0%	1	0.4%	<0.001
	≥ 3 servings a week	904	98.4%	5	0.5%	10	1.1%	
	1-2 servings a week	4017	98.5%	23	0.6%	37	0.9%	
	< 1 serving a week	4764	98.0%	40	0.8%	57	1.2%	
	Rarely or never	9246	90.8%	272	2.7%	662	6.5%	
Dental hygiene	3 o more times a day	7523	97.0%	87	1.1%	148	1.9%	<0.001
	2 times / day	6449	95.7%	99	1.5%	190	2.8%	
	1 time / day	3731	92.5%	93	2.3%	210	5.2%	
	Never or ocasionally	1587	85.1%	60	3.2%	219	11.7%	

Self-perceived health	Very good	4353	99.0%	18	0.4%	26	0.6%	<0.001
	Good	10116	97.1%	114	1.1%	183	1.8%	
	Okay	3651	89.8%	122	3.0%	293	7.2%	
	Bad	966	78.9%	64	5.2%	195	15.9%	
	Very bad	204	68.4%	23	7.5%	72	24.0%	
EuroQol-5D EVA	> 75 points	13132	97.8%	123	0.9%	170	1,3%	<0.001
	50-75 points	4946	91.6%	136	2.5%	319	5.9%	
	< 50 points	1212	77.2%	80	5.1%	278	17.7%	
Some chronic illness	No	11578	97.9%	101	0.9%	144	1.2%	<0.001
	Yes	7712	89.9%	239	2.8%	623	7.3%	
Hypertension	No	15566	96.6%	191	1.2%	362	2.2%	<0.001
	Yes	3724	87.0%	148	3.5%	406	9.5%	
Myocardial infarction	No	19007	95.1%	311	1,6%	679	3.4%	<0.001
	Yes	283	70.7%	29	7.2%	89	22.1%	
Other heart diseases	No	18382	95.6%	301	1.6%	544	2.8%	<0.001
	Yes	908	77.6%	39	3.3%	223	19.1%	
Varicose veins	No	17200	95.1%	287	1.6%	599	3.3%	<0.001
	Yes	2090	90.5%	52	2.3%	168	7.3%	
Arthrosis or arthritis	No	16101	96.6%	209	1.3%	353	2.1%	<0.001
	Yes	3189	85.4%	131	3.5%	414	11.1%	
Chronic neck pain	No	16252	95.3%	259	1.5%	542	3.2%	<0.001
	Yes	3038	90.9%	81	2.4%	225	6.7%	
Chronic low back pain	No	15682	95.5%	243	1.5%	503	3.1%	<0.001
	Yes	3608	90.9%	97	2.4%	264	6.7%	
Chronic allergy	No	16899	94.3%	306	1.7%	716	4.0%	<0.001
	Yes	2391	96.6%	34	1.4%	51	2.1%	
Asthma	No	18337	94.7%	316	1.6%	706	3.6%	<0.001
	Yes	953	91.8%	23	2.2%	61	5.9%	
COPD	No	18552	95.3%	291	1.5%	617	3.2%	<0.001
	Yes	738	78.7%	49	5.2%	151	16.1%	
Diabetes mellitus	No	18092	95.6%	281	1.5%	559	3.0%	<0.001
	Yes	1198	81.8%	58	4.0%	208	14.2%	
Gastrointestinal ulcer	No	18534	94.8%	312	1.6%	699	3.6%	<0.001
	Yes	756	88.7%	28	3.2%	69	8.1%	
Urinary incontinence	No	18815	95.5%	298	1.5%	597	3.0%	<0.001
	Yes	475	69.1%	42	6.1%	170	24.8%	
High cholesterol	No	15828	95.3%	232	1.4%	556	3.3%	<0.001
	Yes	3462	91.6%	107	2.8%	211	5.6%	
Waterfalls	No	18067	96.2%	246	1.3%	466	2.5%	<0.001
	Yes	1223	75.6%	94	5.8%	301	186%	
Skin problems	No	18408	94.7%	319	1.6%	705	3.6%	<0.001
	Yes	882	91.4%	21	2.1%	63	6.5%	
Constipation	No	18588	95.0%	308	1.6%	676	3.5%	<0.001
	Yes	702	85.1%	32	3.8%	92	11.1%	
Cirrhosis	No	19126	94.7%	326	1.6%	743	3.7%	<0.001
	Yes	163	80.8%	14	6.9%	25	12.3%	
Depression	No	17986	95.1%	296	1.6%	636	3.4%	<0.001
	Yes	1304	88.2%	44	3.0%	131	8.9%	
Anxiety	No	17858	94.8%	305	1.6%	673	3.6%	<0.001
	Yes	1431	91.7%	35	2.2%	95	6.1%	
Mental health problems	No	19098	95.0%	326	1.6%	684	3.4%	<0.001
	Yes	192	66.5%	13	4.6%	83	28.9%	

Stroke	No	19133	94.9%	328	1.6%	702	3.5%	<0.001
	Yes	157	67.0%	12	5.1%	65	27.9%	
Migraine	No	17413	94.5%	310	1.7%	695	3.8%	0.736
	Yes	1877	94.9%	29	1.5%	72	3.6%	
Hemorrhoids	No	18199	94.8%	316	1.6%	685	3.6%	<0.001
	Yes	1091	91.2%	24	2.0%	82	6.9%	
Malignant tumor	No	18852	95.1%	260	1.3%	709	3.6%	<0.001
	Yes	438	76.1%	79	13.8%	58	10.2%	
Osteoporosis	No	18606	95.1%	307	1.6%	657	3.4%	<0.001
	Yes	684	82.7%	33	4.0%	110	13.3%	
Thyroid problems	No	18281	94.6%	323	1.7%	720	3.7%	0.499
	Yes	1009	94.1%	16	1.5%	47	4.4%	
Accident injuries	No	18265	94.6%	316	1.6%	719	3.7%	0.201
	Yes	1025	93.4%	24	2.1%	48	4.4%	
Accidents in the last year	No	17700	94.7%	316	1.7%	684	3.7%	0.021
	Yes	1590	93.6%	24	1.4%	84	4.9%	
Mental health	Psychopathology absence	17007	95.6%	265	1.5%	524	2.9%	<0.001
	Suspicion	930	93.6%	20	2.0%	44	4.4%	
	Psychopathology presence	1184	86.6%	49	3.6%	134	9.8%	
	NA	169	70.3%	6	2.6%	65	27.1%	
Limitation of physical activity in previous 2 weeks	No	17266	95.5%	254	1.4%	557	3.1%	<0.001
	Yes	2024	87.2%	86	3.7%	210	9.1%	
Bed rest in previous 2 weeks	No	18346	95.1%	299	1.5%	652	3.4%	<0.001
	Yes	944	85.8%	41	3.7%	115	10.5%	
Limitation of physical activity in previous 6 months	Severely limited	430	64.0%	45	6.7%	197	29.3%	<0.001
	Limited not serious	2866	88.5%	114	3.5%	258	8.0%	
	Nothing limited	15994	97.0%	181	1.1%	312	1.9%	
Glasses or contact lenses	No	7721	97.1%	58	0.7%	174	2.2%	<0.001
	Yes	11569	93.0%	282	2.3%	593	4.8%	
Hearing aids	No	18763	94.9%	323	1.6%	677	3.4%	<0.001
	Yes	527	83.1%	17	2.7%	90	14.2%	
Hospitalization in previous 12 months	No	17855	95.5%	266	1.4%	580	3.1%	<0.001
	Yes	1435	84.6%	73	4.3%	187	11.0%	
Primary care consult in previous month	No	14085	96.0%	190	1.3%	403	2.7%	<0.001
	Yes	5205	91.0%	150	2.6%	364	6.4%	
Visits to other specialists	No	16497	95.2%	250	1.4%	583	3.4%	<0.001
	Yes	2793	91.1%	89	2.9%	184	6.0%	
Stay in day hospital in previous 12 months	No	17901	94.9%	287	1.5%	670	3.6%	<0.001
	Yes	1389	90.3%	53	3.4%	97	6.3%	
Visits to urgencies in previous 12 months	No	14236	95.4%	234	1.6%	456	3.1%	<0.001

	Yes	5054	92.4%	106	1.9%	311	5.7%	
Physiotherapist in previous 12 months	No	17264	94.4%	317	1.7%	716	3.9%	<0.001
	Yes	2026	96.5%	22	1.1%	52	2.5%	
Psychologist in previous 12 months	No	18634	94.6%	333	1.7%	733	3.7%	0.116
	Yes	656	94.1%	7	1.0%	34	4.9%	
Radiography in previous 12 months	No	14173	95.6%	203	1.4%	455	3.1%	<0.001
	Yes	5117	91.9%	137	2.5%	312	5.6%	
CT or scanner in previous 12 months	No	17976	95.3%	255	1.3%	630	3.3%	<0.001
	Yes	1314	85.5%	85	5.5%	137	8.9%	
Ecography in previous 12 months	No	16594	95.0%	243	1.4%	634	3.6%	<0.001
	Yes	2696	92.2%	96	3.3%	133	4.5%	
MRI in previous 12 months	No	17926	94.9%	279	1.5%	679	3.6%	<0.001
	Yes	1364	90.1%	61	4.0%	88	5.8%	
Flu vaccine	No	16123	97.1%	185	1.1%	302	1.8%	<0.001
	Yes	3167	83.6%	154	4.1%	466	12.3%	

CT: computed tomography; MRI: magnetic resonance imaging; NA: not available.
